# Psycho-social predictors of acculturative stress and adjustment in Pakistani Institutions

**DOI:** 10.12669/pjms.35.5.1214

**Published:** 2019

**Authors:** Muhammad Akram Riaz, Rafia Rafique

**Affiliations:** 1 Mr. Muhammad Akram Riaz, MS. Institute of Applied Psychology, University of the Punjab, Lahore, Pakistan; 2 Dr. Rafia Rafique, PHD. Institute of Applied Psychology, University of the Punjab, Lahore, Pakistan

**Keywords:** Psycho-social predictors, Acculturative Stress, Adjustment

## Abstract

**Objectives:**

The main objective of study was to investigate the effect of psycho-social predictors on acculturative stress and adjustment in Pakistani institutions.

**Methods:**

The study was carried out from November 15, 2016 to January 18, 2019. For this purpose data was collected from 450 international students who were studying in public and private sector universities of Pakistan and who experienced acculturative stress. The instruments include Relationship Assessment Scale, Acculturative Stress Scale for International, Psychological Adaptation Scale, and Revised Socio Cultural Adaptation Scale. The proposed model was tested by using SPSS (Version-23) and AMOS (Version-9).

**Results:**

Findings of the study revealed that relationship satisfaction (p<0.05), length of stay (p<0.05), and financial support (p<0.05) significantly negatively predicted acculturative stress. Financial support significantly positively predicted psychological adjustment (p<0.05). Employment status did not predict acculturative stress, psychological and socio-cultural adjustment (p>0.05). Moreover, all psycho-social predictors did not show effect on socio-cultural adjustment among international students (p>0.05).

**Conclusion:**

It can be concluded that general relationship satisfaction, more length of stay in host country, and financial support are very important for international students to manage their acculturative stress and to adjust better in a new cultural environment.

## INTRODUCTION

Acculturative stress is the type of psychological stress the individual experience during cross cultural interaction. Immigrants are very vulnerable to acculturative stress as compared to other normal adults. International students or academic sojourns are such types of immigrants who experience an optimum level of acculturative stress when they study in the country that has different culture than the culture of their own country.^1^ Even those international students who have better cross cultural experience also exhibit some level of acculturative stress. This acculturative stress impacts on the level of adjustment and mental health of immigrant population. Many studies have been conducted on international students’ acculturative stress and adjustment however there is dearth of studies available in the indigenous context.^1^,^2^

The existing body of knowledge is evident that some psycho social predictors including length of stay, financial support, employment status, and relationship with fellows effect on acculturative stress and psychological and socio cultural adjustment of international students. Length of stay has been examined in previous research as an important factor that has impacts on the level of acculturative stress and adjustment.^3^ More length of stay is associated with lower level of acculturative stress and better adjustment in the host country. During cross cultural experience more length of increase linguistic ability and cultural knowledge which is essential for the adjustment process in the host country.^4^

Financial support and employment status also found to be important predictors of acculturative stress and adjustment in the cross cultural experience of international students. International students often have three types of financial supports that may include scholarship, family support or self-support. Students who try to manage their fees and other expenses by self-support have additional stress as compared to those who study on the basis of scholarship.^5^,^6^ International students get debit from banks due to high tuition fesses in host institutes and therefore they search for job to manage their expenses.^7^ Job opportunities and financial support programs for international students are also found to be lacking in host countries.

Lastly, satisfied relationships with fellows is also important factor that can have impact on acculturative stress and adjustment of international students. Interpersonal problems and relationship difficulties increases the level of acculturative stress.^8^ Loneliness was also related to lack of adjustment and higher level of acculturative stress among international students.^9^ These all predictors directly and indirectly impact on the adjustment of international students in host country environment.

Our objective was to investigate the effect of psycho-social predictors on acculturative stress and adjustment in Pakistani institutions

## METHODS

The cross sectional survey research was conducted on 450 international students who experienced acculturative stress from November 15, 2016 to January 18, 2019. Almost all students have some level of acculturative stress and it was confirmed by applying Acculturative Stress Scale for International Students. Data collection was done by using purposive sampling technique. The sample size of 450 participants was representative and sample size estimation was done by Kline criteria of sample size of unknown population. Kline suggested that sample size per parameter for unknown population should be 10 cases per parameter. Moreover he suggested that even for a complex model 450 cases are sufficient sample size for the unknown population.^10^ Data was collected by using following scales; Relationship Assessment Scale is reliable and valid measure used to investigate the relationship satisfaction of the participants. It has seven items and it is likert type scale.^11^ Acculturative Stress Scale for International is reliable and valid measure used to investigate the acculturative stress among international students. It has 36 items and it is likert type scale.^12^ Psychological Adaptation Scale is reliable and valid measure used to investigate the psychological adjustment of international students. It has 10 items and it is likert type scale.^13^ In addition, Revised Socio Cultural Adaptation Scale is reliable and valid measure used to investigate the socio-cultural adjustment of international students. It has 21 items and it is likert type scale.^14^ After taking informed consent questionnaires were distributed to all participants. Each participant took 20-40 minutes in completing questionnaires. Data was collected from six reputed universities of Pakistan where most of international students were studying. These universities include Quaid-i-Azam University Islamabad, International Islamic University Islamabad, National University of Science and Technology Islamabad, National University of Modern Languages Islamabad, University of the Punjab Lahore, and Govt. College University Lahore. The study was approved by the Ethical Committee of Institute of Applied Psychology, University of the Punjab Lahore.

The data was analyzed by using AMOS and SPSS. Structural Equation Modeling (SEM) was conducted by using AMOS (Version-10). Multiple comparisons on socio-demographic characteristics are investigated by using Post Hoc Test (Tucky method) in SPSS (Version-23). The criterion for statistical significance for all tests was p values less than 0.05.

## RESULTS

In the current study data was collected from 450 international students who experienced acculturative stress in which males and females were in equal counterparts with age range 19 to 28 years. The socio-demographic characteristics of participants are shown in Table-I.

**Table I:** Socio-demographic characteristics of the sample.

Number of participants	n	%	Number of participants	n	%
Gender			Country of origin		
Males	225	50	East Asian	106	23.6
Females	225	50	North American	44	9.8
Employment status			Central Asian and Russian States	63	14
Unemployed	396	88.0	Middle East	85	18.9
Employed	54	12.0	East Europe	30	6.7
Financial support			African	107	23.8
Family-support	331	73.6	South Asian	15	3.3
Scholarship	90	20	Chronological age		
Self-support	29	6.4	19 year	15	3.3
Length of stay			20 year	99	22.0
1 year	244	54.2	21 year	111	24.7
2 year	187	41.6	22 year	81	18
3 year	19	4.2	23 year	28	6.2
			24 year	44	9.8
			25 year	25	5.6
			26 year	21	4.7
			27 year	12	2.7
			28 year	14	3.1

**Fig.1:**
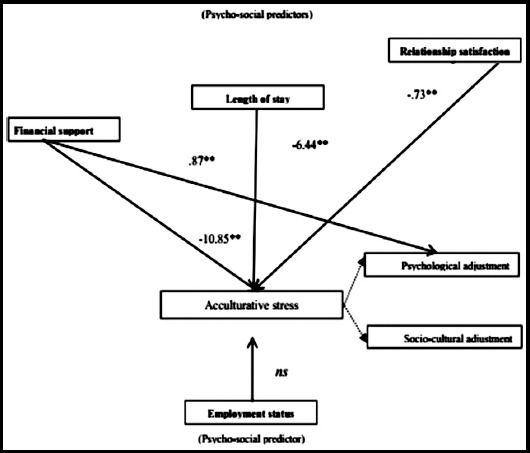
Figures shows the effect of psycho-social predictors of acculturative stress and adjustment.

The psycho-social predictors of acculturative stress, psychological adjustment and socio cultural adjustment are shown in Table-II. Results show that relationship satisfaction significantly negatively predicted acculturative stress (β=0.73, p<0.001); length of stay significantly negatively predicted acculturative stress (β=6.44, p<0.001) and financial support also significantly negatively predicted acculturative stress (β=10.85, p<0.001) whereas significantly positively predicted psychological adjustment (β=0.87, p<0.001) among international students. Employment status did not predict acculturative stress, psychological adjustment and socio-cultural adjustment. The tested model based on psycho-social predictors of acculturative stress, psychological adjustment and socio cultural adjustment is shown in Fig.1.

**Table II:** Psycho-social predictors of acculturative stress, psychological adjustment and socio cultural adjustment.

Variables	Acculturative stress	Psychological adjustment	Socio-Cultural adjustment
Relationship satisfaction	-.73**	ns	ns
Length of stay	-6.44**	ns	ns
Financial support	-10.85**	.87**	ns
Employment status	ns	ns	ns

Table-III shows Post Hoc Test for exploring multiple differences on length of stay and financial support and its impacts on acculturative stress and psychological adjustment. It was found that international students who were staying not more than one year in Pakistan significantly scored higher on acculturative stress (MD=80.53, p<0.05). On financial support results show that international students who were on self-support significantly scored higher on acculturative stress (MD=56.89, p<0.05). On the other hand those international students who had scholarship significantly scored higher on psychological adjustment (MD=4.23, p<0.05).

**Table III:** Post Hoc Test for exploring multiple differences on length of stay and financial support and its impacts on acculturative stress and psychological adjustment.

Dependent Variable	(I) Length of stay	(J) Length of stay	MD (I-J)	P value	95% CI	

					Lower	Upper
Acculturative Stress	2 years	1 year	80.53^*^	.00	74.55	86.52
Dependent Variables	(I) Scholarship Self-support	(J) Scholarship Self-support	MD (I-J)	p	95% CI	
					LL	LL
Acculturative stress	Scholarship	Self-support	56.89^*^	.00	45.26	68.52
Psychological adjustment	Family support	Scholarship	4.23^*^	.00	3.31	5.16

## DISCUSSION

In the current study a behavioral model is tested that was based on psycho-social predictors of acculturative stress and adjustment among international students. It was found that relationship satisfaction, length of stay, and financial support significantly negatively predicted acculturative stress. Financial support significantly positively predicted psychological adjustment among international students. However these psycho-social predictors did not show effect on socio-cultural adjustment which means that these psycho-social predictors are more important for the psychological adjustment rather than socio-cultural adjustment of international students. Our study further confirmed that higher the length of stay was associated with lower level of acculturative stress. In a study, Ayoob et al. investigated differences in length of stay is related with acculturative stress and health.^15^ Similarly better relationship satisfaction and financial support also associated with lower acculturative stress which creates better psychological adjustment.^16^,^17^ Previous studies also supported these findings that those international students who have better relationships with family and host fellows had lower level of acculturative stress.^18^,^19^ Irum, Ajmal, and Sabah found that disrespectable attitude from host fellows also creates acculturative stress among international students.^20^ Those international students who study on family support or scholarship also found to be lower on acculturative stress.^21^ In a more recent study, Lam investigated culture shock (acculturative stress) and found that supportive social network and individual personality factors are essential to manage the effects of culture shock.^22^ Moreover, it was found that employment status did not predict acculturative stress, psychological adjustment and socio-cultural adjustment. Some previous studies have showed the effect of employment status on acculturative stress and adjustment.^23^,^24^ The current findings are unique, non-significant effect of employment status may be due to that most of the international students do not expect to get employment in Pakistan because Pakistan is under developing country and most of international students who came to Pakistan are from well off countries of middle East and East Asia.

### Limitations and suggestions

The current study was based on cross sectional survey design, it will more better if a longitudinal study will be conducted in which researcher will able to investigate that how psycho-social factors develop acculturative stress and how international students manage these factors and reach at the point of adjustment. But in the current study due to time and resources constrains it was not possible to conduct a longitudinal study. Moreover, if researcher investigated acculturative stress among international students and other immigrant population then we will able to investigate that which type of immigrants experience higher level of acculturative stress. It is recommended that future research should also investigate international students and other types of immigrants’ problems consecutively.

## CONCLUSION

Based on the current study it can be concluded that general relationship satisfaction, higher stay in host country and better financial support is significantly important for the international students’ management of acculturative stress as well as for better psychological adjustment in host institution. The current study has implication for health professionals, psychologists and other clinicians who are working for the betterment of physical and mental health of international students.

### Authors Contribution

**MAR:** Conceived, designed, data collection and did statistical analysis.

**RR:** Did review, manuscript writing, editing of manuscript and final approval of manuscript.
